# The past, present and future of high-performance fibers

**DOI:** 10.1093/nsr/nwae310

**Published:** 2024-09-04

**Authors:** Zhenzhen Liu, Yan Wang, Junrong Yu, Yinjun Chen, Meifang Zhu

**Affiliations:** State Key Laboratory for Modification of Chemical Fibers and Polymer Materials, College of Materials Science and Engineering, Donghua University, China; State Key Laboratory for Modification of Chemical Fibers and Polymer Materials, College of Materials Science and Engineering, Donghua University, China; State Key Laboratory for Modification of Chemical Fibers and Polymer Materials, College of Materials Science and Engineering, Donghua University, China; State Key Laboratory for Modification of Chemical Fibers and Polymer Materials, College of Materials Science and Engineering, Donghua University, China; State Key Laboratory for Modification of Chemical Fibers and Polymer Materials, College of Materials Science and Engineering, Donghua University, China

## Abstract

This paper summarizes the recent progress of diverse high-performance fibers in their properties, applications and the challenges.

High-performance fibers currently play an indispensable role in aerospace, national defense military, rail transportation, renewable energy and sports equipment because of their light weight and exceptional properties, including high strength and modulus, excellent high-temperature and radical resistance, superb chemical stability, etc. High-performance fibers mainly include carbon fibers, aramid fibers, ultra-high molecular weight polyethylene (UHMWPE) fibers, basalt fibers, polyphenylene sulfide fibers, polyimide fibers and poly(*p*-phenylene benzobisoxazole) (PBO) fibers, etc. [1]. Their outstanding properties endow sophisticated equipment with high performance, weight reduction, high degrees of protection and reliability, which accelerate exploration in deep space and sea. However, with the rapid advancement of technology, higher and harsher requirement is put forward for high-performance fibers to meet the demands of diverse fields. In consequence, new categories of high-performance fibers are continually being developed. Herein, we summarize the recent progress in high-performance fibers regarding properties, application areas and challenges, and propose future perspectives.

## CARBON FIBERS

**Figure 1.**
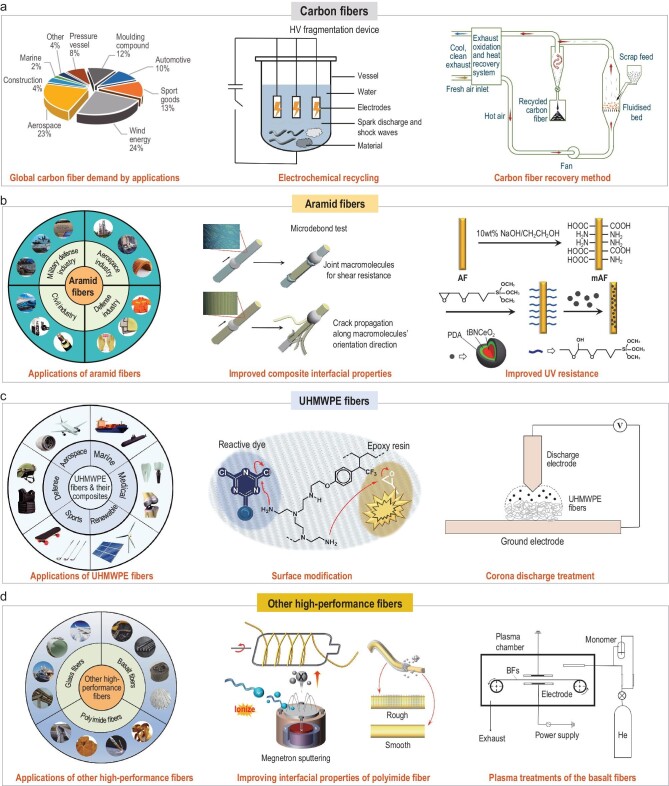
Application and optimized methods of high-performance fibers. (a) Global carbon fibers demand in application and recycling strategies (redrawn from [3]). (b) Application of aramid fibers and current methods to improve their properties (redrawn from [4]). (c) Application of UHMWPE fibers and current approaches to improve their properties (redrawn from [7,8]). (d) Other types of high-performance fibers (redrawn from [10]).

Carbon fibers composed of >90% carbon are characterized by their light weight, high strength and modulus, corrosion resistance, freedom from creep, excellent electrical and thermal conductivity, exceptional resistance to ultra-high temperatures and good fatigue resistance. They serve as crucial basic materials in high-tech industries such as aerospace, renewable energy and high-end equipment manufacturing. Additionally, they are essential materials for the production of rockets, missiles, fighter jets, naval ships and various cutting-edge military weapons. However, as technology continually advances and application demands increase, carbon fibers are facing certain challenges. (i) High cost and complex processes: expensive raw materials and complicated manufacturing procedures of carbon fibers lead to a stubbornly high cost. Therefore, the need for a simple, low-cost and efficient production process of carbon fibers is pressing. In this regard, commercial low-cost plastics, including polyethylene, polyamides, polystyrene and polyesters, have recently been used as precursors for the synthesis of carbon fibers, replacing expensive polyacrylonitrile. (ii) Difficulty in recycling: the widespread use of carbon fibers results in considerable waste, posing significant environmental challenges. Currently, the primary recycling technology is pyrolysis—a mature procedure that is applicable to all stages of carbon fiber composites [2]. (iii) Energy consumption: the production of carbon fibers is an energy-intensive process, consuming 198–595 MJ/kg of energy, which is ∼10 times that of other synthetic fibers [3]. (iv) Stagnated iteration: aviation-grade carbon fibers such as IM7 and T800H were developed 30 years ago, while there is little improvement in the tensile and compressive strength of carbon fibers to date. Continuous efforts should be made to update the mechanical properties of carbon fibers and address strength deficiencies (Fig. 1a).

## AROMATIC POLYAMIDE FIBERS

Aramid fibers (AFs), including meta-aramid, para-aramid and heterocyclic aramid fibers, are aromatic polyamide fibers. Among them, para- and heterocyclic AFs have excellent mechanical properties, as well as high-temperature resistance, insulation and flame retardancy, light weight and good chemical stability. AFs are widely used in weapons industry, bulletproof armor, optical cable reinforcement, high-temperature protection, automotive industry, aerospace, leisure sports, electrical industry and composite materials [4]. However, there are still some unresolved issues regarding the properties and productive processes of AFs. (i) Inert chemical surface and undesired ultraviolet (UV) irradiation resistance: AFs degrade under ultraviolet radiation, significantly limiting their application in long-term outdoor environments, especially under harsh aerospace conditions. Currently, the strategies for enhancing the UV resistance primarily focus on applying UV-absorbing/shielding inorganic coatings, by *in situ* chemical grafting, sol–gel, hydrothermal reduction, hydrothermal crystallization, copolymerization, layer-by-layer self-assembly and atomic layer deposition. However, these methods significantly increase the weight of AFs and severely compromise their flexibility and mechanical properties, and may even accelerate the aging process [5]. (ii) Water and moisture absorption: AFs exhibit a relatively high hygroscopicity (≤6 wt%). Therefore, aramid-fiber-reinforced composites normally require appropriate protection to reduce moisture absorption. (iii) High-pollution manufacturing technique: most solvents used in the preparation of AFs are toxic and environmentally unfriendly. Meanwhile, the manufacturing facility and technique are expensive and sophisticated, leading to the high cost of AFs. Consequently, more efforts should be made to develop a simple, efficient, cost-effective and environmentally friendly manufacturing technique by developing novel monomers and eco-friendly solvents (Fig. 1b).

## UHMWPE FIBERS

UHMWPE fibers exhibit numerous exceptional properties, including lightness, high strength and modulus, as well as good chemical resistance and abrasion resistance. In consequence, UHMWPE fibers have been widely used in protective devices (bulletproof vests and helmets, cut-resistant gloves, etc.), net cages, fishing nets and rope [6]. However, surface inertia, poor creep resistance and inferior high-temperature resistance restrict the application of UHMWPE fibers. At present, plasma treatment, corona discharge, silane cross-linking, UV-induced grafting and cross-linking, chemical oxidation and coating treatment are performed to ameliorate these drawbacks [7], although these approaches often result in a decrease in fiber strength. Therefore, it is crucial to seek a new method that overcomes the drawbacks of UHMWPE fibers without compromising their strength. Recently, with the aim of expanding the civilian application of UHMWPE fibers, research on their differential modification has also attracted great attention [8]. In summary, the application prospects for UHMWPE fibers remain vast in spite of their disadvantages. Future research will concentrate more on performance optimization, new application development and sustainability (Fig. 1c).

## OTHER CATEGORIES OF HIGH-PERFORMANCE FIBERS

For applications in various extreme environments, a wide variety of fibers with novel and improved performance are constantly springing up. For instance, polyimide fibers exhibiting a broader temperature resistance range (–267 to 550°C) have been developed to offer superior heat and weather resistance. Cross-linked basalt fibers possess excellent acid and alkali resistance, low- and high-temperature resistance (usable between –260 and 700°C) and remarkable wettability [9]. Due to their 3D molecular structure, cross-linked basalt fibers present significantly improved impact resistance, tensile strength and toughness. Additionally, glass fibers, characterized by low weight, high strength, cost-competitiveness, superior insulation and corrosion resistance, dominate the fiber reinforcement segment of the composite materials industry, occupying >95% of the market share. Other new types of high-performance fibers, including silicon carbide fibers, polybenzimidazole fibers and PBO fibers, have been successively developed.

While one would imagine that more high-performance fibers will be developed, the continuous optimization of fibrous properties and cost-effectiveness will be the key points along that line. Additionally, multifunctional and ‘intelligent’ fibers will be considered to meet more diverse applications. Last, the degradability and recyclability of high-performance fibers post-lifespan will be another future theme (Fig. 1d).

## References

[1] Gleissner C, Landsiedel J, Bechtold T et al. Polym Rev 2022; 62: 757–88. 10.1080/15583724.2022.2025601

[2] Meng F, Olivetti EA, Zhao Y et al. ACS Sustain Chem Eng 2018; 6: 9854–65. 10.1021/acssuschemeng.8b01026

[3] Zhang J, Chevali VS, Wang H et al. Compos Part B-Eng 2020; 193: 108053. 10.1016/j.compositesb.2020.108053

[4] He A, Xing T, Liang Z et al. Adv Fiber Mater 2023; 6: 3–35. 10.1007/s42765-023-00332-1

[5] Dong L, Shi M, Xu S et al. RSC Adv 2020; 10: 22578–85. 10.1039/D0RA03120H 35514588 PMC9054610

[6] Vlasblom M . Handbook of Properties of Textile and Technical Fibres. Amsterdam: Elsevier, 2018.

[7] Shelly D, Lee S-Y, Park S-J. Compos Part B-Eng 2024; 275: 111294. 10.1016/j.compositesb.2024.111294

[8] Nazir R, Musolino SF, MacFarlane MA et al. ACS Appl Polym Mater 2024; 6: 1688–97. 10.1021/acsapm.3c02531

[9] Zheng Y, Zhang Y, Zhuo J et al. Constr Build Mater 2022; 359: 129360. 10.1016/j.conbuildmat.2022.129360

[10] Kim S-H, Lee J-H, Kim J-W et al. Adv Fiber Mater 2022; 4: 1414–33. 10.1007/s42765-022-00204-0

